# Syndrome de démyélinisation osmotique compliquant la correction rapide d’une hyponatrémie sévère associée à une hypokaliémie

**DOI:** 10.11604/pamj.2019.34.208.19968

**Published:** 2019-12-20

**Authors:** Zakaria Ghoummid, Mbark El Kaouri, Mina Elkhayari

**Affiliations:** 1 Service d’Accueil des Urgences Vitales, Département d’Anesthésie Réanimation et Urgences, Hôpital Ibn Tofail, CHU Mohammed VI, Faculté de Médecine et de Pharmacie de Marrakech, Université Cadi Ayyad, Marrakech, Maroc

**Keywords:** Syndrome de démyélinisation osmotique, myélinolyse centropontine, myélinolyse extrapontine, hypnatrémie, hypokaliémie, imagerie par résonance magnétique, Osmotic demyelination syndrome, centropontine myelinolysis, extrapontine myelinolysis, hyponatremia, hypokalemia, magnetic resonance imaging

## Abstract

Le syndrome de démyélinisation osmotique correspond à une démyélinisation du centre de la protubérance ou d'autres parties du cerveau. Nous rapportons un cas de syndrome de démyélinisation osmotique chez une patiente âgée de 55 ans, connue hypertendue, suivie pour cervicarthrose étagée, hospitalisée pour coma non fébrile d'installation brutale compliquant un tableau de gastroentérite aigue. L'évolution a été marquée par l'aggravation neurologique avec confusion, aphasie, tétraplégie et aréflexie ostéotendineuse. Le diagnostic de myélinolyse centropontine et extrapontine a été confirmé par une imagerie par résonance magnétique cérébrale faite 20 jours après une première qui n’a pas montré d'anomalies spécifiques. La correction rapide de l'hyponatrémie était la cause principale de ce syndrome, sans négliger le rôle favorisant très probable de l'hypokaliémie associée. L'évolution de la myélinolyse centropontine (MCP) et myélinolyse extrapontine (MEP) est variable. Le traitement est principalement préventif basé sur la correction prudente des hyponatrémies sévères et des facteurs favorisants.

## Abstract (en)

Osmotic demyelination syndrome is characterized by the loss of myelin in the center of the basis pontis and other areas of the central nervous system. We report a case of osmotic demyelination syndrome in a 55-year-old female, with a past medical history of arterial hypertension and multi-level cervical spondylosis, hospitalized for acute altered mental status complicating an array of acute gastroenteritis, the patient was afebrile. The course was marked by neurologic aggravation with confusion, aphasia, tetraplegia and osteo-tendinous areflexia. Initial cerebral magnetic resonance imaging did not show any specific abnormalities. The diagnosis of Central pontine myelinolysis and extrapontine myelinolysis was confirmed by a cerebral magnetic resonance imaging done after 20 days of the first. The rapid correction of hyponatremia was the main cause of this syndrome, without neglecting the very likely role of the associated hypokalemia. The evolution of centropontine myelinolysis (CPM) and extrapontine myelinolysis (EPM) is variable. The treatment is primarily preventive based on the careful correction of severe hyponatraemia and contributing factors.

## Introduction

La myélinolyse centropontine et extrapontine (MCP, MEP) ou syndrome de démyélinisation osmotique (SDO) est une pathologie rare, caractérisée par la destruction des gaines de myéline [1]. La physiopathologie précise de ce syndrome est encore mal connue. La myélinolyse centropontine a été décrite pour la première fois en 1959 par Adams et ses collègues comme une pathologie affectant les patients alcooliques et les dénutris [2]. L'association des lésions de MCP et MEP est fréquente et pourrait atteindre jusqu'à 30% [3]. La correction trop rapide d'une hyponatrémie reste la cause la plus classique de ce syndrome [4]. Dans la littérature, le développement du SDO peut être lié à de nombreux facteurs prédisposants comme la sécrétion inappropriée de l'hormone antidiurétique, l'alcoolisme chronique, la malnutrition, la polydipsie psychogène, la transplantation hépatique, la dialyse ect. [5]. D'autres facteurs comme l'hypokaliémie semblent jouer aussi un rôle favorisant [4]. Nous rapportons une observation de MCP et MEP survenant après la correction rapide d'une hyponatrémie sévère associée à une hypokaliémie.

## Patient et observation

**Figure 1:**
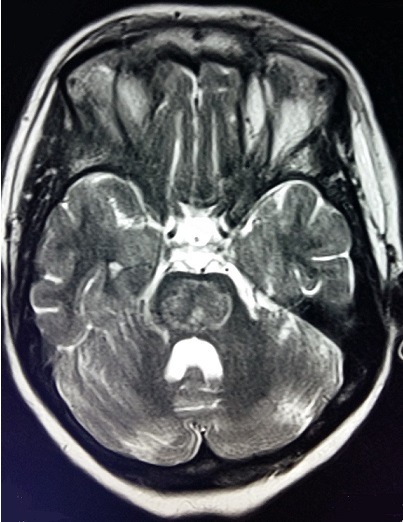
IRM cérébrale en coupe axiale montrant un hypersignal en pondération T2 au niveau de la protubérance en faveur d’une myélinolyse centropontine

**Figure 2:**
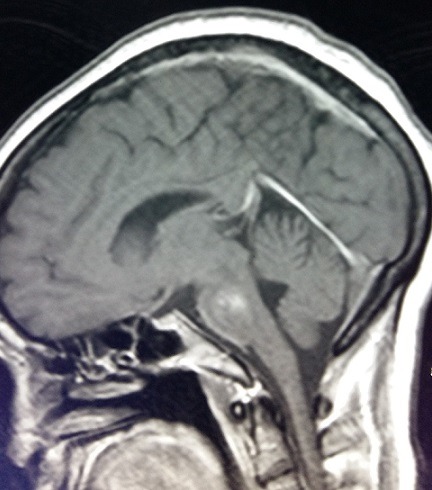
IRM cérébrale en coupe sagittale montrant un hypersignal en séquence flair au niveau de la protubérance en faveur d’une myélinolyse centropontine

**Figure 3:**
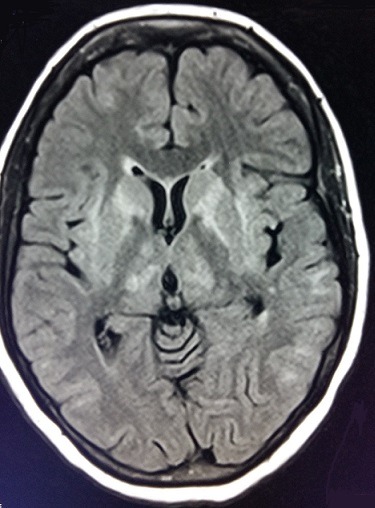
IRM cérébrale en coupe axiale montrant un hypersignal en séquence flair au niveau du cortex et des noyaux gris centraux en faveur d’une myélinolyse extrapontine

Nous rapportons le cas d'une patiente âgée de 55 ans, connue hypertendue sous antagonistes des récepteurs de l'angiotensine II, suivie pour cervicarthrose étagée sous anti-inflammatoires non stéroïdiens. Admise initialement aux urgences pour coma non fébrile d'installation brutale compliquant un tableau de gastroentérite aigue évoluant depuis 48 heures. Le bilan biologique montrait une hyponatrémie sévère à 92mmol/l associée à une hypokaliémie à 2.7 mmol/l. Après une correction initiale au service d'accueil des urgences vitales avec du sérum salé isotonique à une vitesse non précisée, la natrémie a passé à 118mmol/l après 24h d'hospitalisation (soit une augmentation de 26mmol/l en 24h). La tomodensitométrie cérébrale était normale. Deux jours après cette correction rapide de l'hyponatrémie avec une vitesse dépassant 1mmol/l/h, la patiente a été transférée en réanimation médicale pour continuer sa prise en charge. L'examen clinique trouvait une patiente consciente, stable sur le plan hémodynamique et respiratoire avec dysarthrie et tétraparésie. Un ionogramme sanguin de contrôle a montré une natrémie à 122mmol/l avec augmentation de la kaliémie à 3.4mmol/l. L'évolution a été marquée par l'aggravation neurologique avec confusion, aphasie, tétraplégie et aréflexie ostéotendineuse. Une imagerie par résonance magnétique (IRM) encéphalique a été faite à J11 d'hospitalisation montrant des anomalies de signal punctiformes de la substance blanche sus-tentorielle de nature non spécifique. Devant l'aggravation neurologique et la persistance d'une hypokaliémie modérée et d'une hyponatrémie variant entre 122mmol/l et 132mmol/l malgré une correction qui ne dépassait pas 1mmol/l/h, un bilan paraclinique poussé était réalisé, montrant une hyperthyroïdie (TSH basse à 0,07mUI/l, T4 élevée à 27pmol/l), une cortisolémie à 182ng/ml, un ionogramme urinaire normal, une étude du liquide céphalorachidien normale, un électromyogramme était en faveur d'une polyneuropathie axonale, et un électroencéphalogramme montrant de multiples foyers de souffrance cérébrale. L'IRM cérébrale refaite à J31 d'hospitalisation montrait une MCP et MEP (Figure 1, Figure 2, Figure 3). Le prise en charge consistait en la poursuite de la correction de l'hyponatrémie, avec une vitesse de 0,5mmol/l/h avec contrôle quotidien, kinésithérapie quotidienne respiratoire et rééducation motrice, alimentation par voie entérale, protection gastrique et thromboembolique. L'évolution était défavorable, marquée par le décès de la patiente après un mois et demi d'hospitalisation suite à un choc septique à point de départ pulmonaire.

## Discussion

La myélinolyse centropontine et extrapontine se regroupent sous le terme de syndromes de démyélinisation osmotique (SDO) [6]. L'incidence réelle de cette pathologie est inconnue jusqu'à ce jour [7]. Microscopiquement, il s'agit d'une destruction symétrique de la myéline qui intéresse tous les faisceaux nerveux associée à une perte des oligodendrocytes [2]. Dans la MCP, une étude a montré la présence de lésions axonales importante associées à un infiltrat inflammatoire [8]. La correction rapide d'un désordre osmolaire d'installation chronique en cas de déficit en osmolytes organiques expose les cellules cérébrales particulièrement les oligodendrocytes au risque de rétrécissement et par conséquence à la démyélinisation [9]. Dans une étude de 22 cas présentant une hyponatrémie, l'hypokaliémie s'est révélée être un facteur prédisposant dans 7 cas de MCP [10]. En cas d'hypokaliémie, la diminution de la concentration de NaK-ATPase dans la membrane cellulaire endothéliale peut prédisposer la cellule à des lésions par le stress osmotique associé à l'augmentation rapide de la natrémie [11]. Probablement l'hypokaliémie associée dans notre cas a aussi joué un rôle favorisant.

Le diagnostic de la MCP et MEP est essentiellement clinique, la symptomatologie suit généralement une évolution biphasique. Dans un premier temps le patient peut présenter une encéphalopathie qui peut se rétablir rapidement après restauration de la natrémie. Plusieurs jours plus tard, la seconde phase est caractérisée par l'apparition d'une dysarthrie, dysphagie secondaires à l'atteinte cortico-bulbaire et d'une quadriparésie flasque devenant ensuite spastique secondaire à l'atteinte cortico-spinale [6]. Dans notre cas, l'évolution était aussi biphasique, ce qui rejoint la littérature. La TDM cérébrale est peu sensible pour le diagnostic [12]. L'IRM cérébrale est l'examen radiologique de choix, elle montre une plage en hypersignal sur les séquences pondérées T2 et FLAIR, en hyposignal en pondération T1 [13]. L'apparition de ces anomalies est habituellement retardée, elle est de l'ordre de 10 à 15 jours, une IRM initialement normale n'élimine pas le diagnostic [14]. L'étude du LCR est le plus souvent normale, mais une hyperprotéinorachie peut être présente [15, 16]. L'EEG, non spécifique, montre le plus souvent un ralentissement diffus de l'activité cérébrale [15]. Les données de la littérature varient considérablement en ce qui concerne la mortalité, allant de 6% à 90% [3, 17]. Le traitement est essentiellement préventif basé sur une correction raisonnée et progressive de toute hyponatrémie, sans dépasser une vitesse de correction de 0,5 mmol/l/h [18]. Il existe d'autres formules récentes de correction, notamment celle de d'Adrogué et Madias qui aide considérablement comme moyen thérapeutique dans toutes les situations de dysnatrémie [19]. Jusqu'à ce jour aucun traitement curatif n'a été codifié.

## Conclusion

L'évolution de la MCP et MEP est variable, souvent défavorable. Le traitement est principalement préventif basé sur la correction simultanée et prudente des hyponatrémies sévères et des autres facteurs favorisants, y compris l'hypokaliémie. Les recherches actuelles se concentrent sur l'étude de l'efficacité de certaines options thérapeutiques notamment les échanges plasmatiques et les immunoglobulines intraveineuses qui ont montré leur efficacité dans certains cas isolés.

## Conflits d’intérêts

Les auteurs ne déclarent aucun conflit d'intérêts.
